# Silicon Improves Rice Salinity Resistance by Alleviating Ionic Toxicity and Osmotic Constraint in an Organ-Specific Pattern

**DOI:** 10.3389/fpls.2020.00260

**Published:** 2020-03-12

**Authors:** Guochao Yan, Xiaoping Fan, Miao Peng, Chang Yin, Zhuoxi Xiao, Yongchao Liang

**Affiliations:** Ministry of Education Key Laboratory of Environment Remediation and Ecological Health, College of Environmental and Resource Sciences, Zhejiang University, Hangzhou, China

**Keywords:** silicon, salinity, rice (*Oryza sativa* L.), osmotic constraint, ionic toxicity

## Abstract

Salinity stress severely inhibits the growth of plant via ionic toxicity and osmotic constraint. Exogenous silicon (Si) can alleviate salinity stress, but the mechanisms behind remain unclear. To investigate the role of Si in alleviating ionic and osmotic components of salinity, rice (*Oryza sativa* L.) seedlings were grown hydroponically in iso-osmotic stress conditions developed from NaCl or polyethylene glycol (PEG). The effects of Si on the growth of shoot and root of rice under salinity and PEG-derived osmotic stress were evaluated and further compared using principal coordinate analysis (PCoA). We also analyzed the concentrations of Na, K, and compatible osmolytes, tissue sap osmotic potential, antioxidant enzymes activities, and the expression of aquaporin genes. Generally, Si significantly promoted shoot and root growth in rice exposed to both NaCl and PEG. PCoA shows that the Si-induced distance change under NaCl treatment was larger than that under PEG treatment in the shoot, while the Si-induced distance changes under NaCl and PEG treatments were at an equal level in the root. Under salinity, Si decreased Na concentration and Na/K ratio in the shoot but not in the root. However, Si decreased net Na uptake and increased root Na accumulation content. Osmotic potential was increased in the shoot but decreased in the root by Si addition. Si decreased soluble sugar and proline concentrations in the shoot but increased soluble sugar and soluble protein concentrations in the root. Besides, Si promoted shoot transpiration rate and root morphological traits. Although both NaCl and PEG treatments upregulated aquaporin gene expression, Si addition maintained the expression of *OsPIP*s under NaCl and PEG treatments at same levels as control treatment. Furthermore, Si alleviated oxidative damages under both NaCl and PEG by regulating antioxidant enzyme activities. In summary, our results show that Si improves salt stress tolerance in rice by alleviating ionic toxicity and osmotic constraint in an organ-specific pattern. Si ameliorates ionic toxicity by decreasing Na uptake and increasing root Na reservation. Si alleviates osmotic constraint by regulating root morphological traits and root osmotic potential but not aquaporin gene expression for water uptake, and promoting transpiration force but not osmotic force in shoot for root-to-shoot water transport.

## Introduction

Silicon (Si) is the second most abundant element in soil and a widely recognized beneficial element in plant (Epstein, 1994, 1999; Liang et al., 2015). The alleviation effect of Si on salinity stress, one of the major environmental problems, has been found in different kinds of plant species including rice, barley, wheat, sorghum, tomato, and cucumber (Liang et al., 2003; Zhu et al., 2004; Gong et al., 2006; Hattori et al., 2007; Saqib et al., 2008; Shi et al., 2016). Salinity stress limits crop production severely and causes inestimable economic loss globally. The situation is getting worse due to global warming and excess chemical fertilization (Munns and Gilliham, 2015). Under such a background, the application of Si fertilizer would be a promising approach with the advantages of high efficiency and low economic cost (Liang et al., 2015; Yan et al., 2018). However, the veiled mechanism behind Si-induced salt stress tolerance impedes the extensive application of Si fertilizer in agriculture.

When grown under salinity stress, plants suffer from two stress components: osmotic constraint and ionic toxicity (Munns and Tester, 2008). Excess salt ions (mainly Na) in growth substance would cause osmotic potential decline, which limits root water uptake and induces successive tissue dehydration (Munns, 2002). The accumulation of salt ions in plant tissues, especially in photosynthetic organ, would increase inevitably over time and successively disrupt plant Na/K balance and essential metabolism (Zhu, 2001; Kronzucker and Britto, 2011; Wakeel et al., 2011). In addition, both ionic and osmotic components of salinity can induce reactive oxygen species accumulation and membrane peroxidation (Munns, 2002; Munns and Tester, 2008). In rice, Si has been found to play a role as physical barrier in the root and block Na apoplastic transport pathway (Gong et al., 2006). Si addition can also improve water use efficiency in rice seedlings under salt stress (Shi et al., 2013). In sorghum, Si can decrease shoot Na concentration by regulating polyamine metabolism (Yin et al., 2016) and enhance root water hydraulic conductance and plant water uptake when grown under salinity (Liu et al., 2015). Si was also found to promote the ability of Na compartmentation and the nutrient status in barley under salt stress (Liang et al., 2005b). In cucumber grown under saline condition, Si protects plant from salinity-induced water deficit and ionic toxicity by decreasing Na uptake, regulating compatible solutes metabolism, and improving root water uptake (Wang et al., 2015; Zhu et al., 2015, 2016). Altogether, Si can improve plant salt tolerance by alleviating both ionic toxicity and osmotic constraint at the same time. Therefore, a better understanding of the contribution of Si-induced ionic and osmotic tolerance improvement to plant growth under salt stress and the expatiating on the associated mechanisms would be key issues to get the whole picture of the beneficial roles of Si under salinity.

Iso-osmotic growth conditions would be an effective method to discriminate the relative contribution of Si-induced ionic detoxification and osmotic stress alleviation to elucidate the underlying mechanisms. Upon dissecting the outcomes of researches aiming to distinguish the ionic effect from osmotic components on plant growth without Si addition, we know that a comparison between the effects of salinity and iso-osmotic conditions developed from non-penetrant chemicals (e.g., polyethylene glycol, PEG) would be suitable. The ionic effect of salinity can be isolated by subtracting the osmotic effect from the total effects of salinity (Lefevre et al., 2001; Munns, 2002). In addition, such comparison has been successfully conducted in rice, wheat, and soybean to distinguish the plant adaptations to ionic and osmotic components of salinity with respect to seed germination, plant growth, proline concentration, polyamine metabolism, K homeostasis, and gene expression (Shabala, 2000; Almansouri et al., 2001; Lefevre et al., 2001; Umezawa et al., 2002; Ahmad et al., 2007).

Rice is one of the most salt-sensitive cereals and a major crop accounting for more food supply than any other crop. Besides, rice is a typical Si accumulator in which the Si transport system has been well-investigated. Thus, rice can benefit from Si addition at a relatively high level (Ma and Yamaji, 2015; Yan et al., 2018). In this work, rice seedlings were grown under iso-osmotic stress conditions developed from NaCl or PEG with or without Si addition to (i) distinguish the growth promotion effects of Si in response to salinity-induced osmotic constraint and ionic toxicity in the shoot and root and (ii) investigate the mechanisms behind Si-induced salinity stress alleviation.

## Materials and Methods

### Plant Growth and Treatment Application

Rice (*Oryza sativa* L. cv. Zhonghua11, ZH11) seeds were sterilized with 10% (*v*/*v*) H_2_O_2_ and then washed five times with deionized water. Seeds of rice were germinated on moist filter paper and then sown on sterilized quartz sand. After 5 days of growth, each 10 rice seedlings were transplanted to a plastic container filled with 3 L half-strength Kimura nutrient solution prepared as described by Liang et al. (2006), and the nutrient solution was renewed every 3 days. The experiment was conducted in a controlled-environment growth chamber with the temperature regime of 25 ± 5°C, humidity regime of 50 ± 5% and 14/10 h light/dark period.

Uniform rice seedlings (28 days old) were used for treatment application. NaCl and PEG (average molecular weight, 6,000) were added with the final concentrations of 100 mM and 18% *(w*/*v*), respectively. For a better discrimination of the effects of Si in response to ionic and osmotic components of salt stress, 100 mM NaCl was used in this study (Lefevre et al., 2001). The osmotic potential of nutrient solution was measured with a dew point osmometer (5600, Wescor, United States). NaCl and PEG addition caused osmotic potential decline of −0.472 and −0.481 MPa, respectively. Si was added in the form of silicic acid, and the final concentration of Si was 1.5 mM. Silicic acid was prepared by passing sodium silicate solution through H^+^-form cation exchange resin (Amberlite IR120, Sigma, United States) according to Liang et al. (2005a). After a 5-day treatment, rice seedlings were harvested with root and shoot separated.

### Plant Dry Weight and Root Morphological Traits

Dry weight (DW) was determined after oven drying at 105°C for 1 h and at 80°C for 72 h. For root morphological traits analysis, fresh root samples were spread in a water layer immediately and scanned with a root scanner (GXY-A, Top Instrument, China). Total root surface area, total root length, and root number (diameter > 0.5 mm) were analyzed using the software compatible to the root scanner.

### Chlorophyll Concentration, Photosynthesis Rate, and Transpiration Rate

Chlorophyll was extracted with 95% (*v/v*) ethanol and measured at 665, 649, and 470 nm using an ultraviolet–visible (UV-vis) spectrophotometer (Evolution 201, Thermo Fisher, United States). Chlorophyll concentration was calculated with the formula described by Porra et al. (1989). The second youngest fully expanded leaf was used for photosynthesis rate (*Pn*) and transpiration rate (*Tr*) measurement with a portable photosynthesis system (6400, Licor, United States). *Pn* and *Tr* were calculated according to the leaf surface area in the test chamber.

### Relative Water Content

Relative water content (RWC) was measured following the procedure described by Weatherley (1950). Briefly, fresh weight (FW) of each sample was measured immediately after harvest. Then, the samples were incubated in tubes containing 10 ml water on a shaker at 30°C for 6 h. After the measurement of turgid weight (TW), samples were dried at 70°C for 72 h, and then dry weight (DW) was determined. RWC was calculated as (FW - DW)/(TW - DW).

### Na and K Concentrations

Dry shoot and root samples were ground into fine powder and digested in 5 ml HNO_3_ and 1 ml H_2_O_2_ with a microwave digest system (Jupiter, Sineo, China). The concentrations of Na and K were measured with a flame photometer (FP640, Inesa Instrument, China).

### Oxidative Damages

For malondialdehyde (MDA) concentration measurement, rice samples were homogenized with 0.1% (*w/v*) trichloroacetic acid solution. After centrifugation at 10,000 rpm for 10 min, the supernatant was mixed with 0.5% (*w/v*) thiobarbituric acid solution and incubated at 96°C for 20 min. Then, the reaction tubes were centrifuged at 10,000 rpm for 10 min. The absorbance of the supernatant was measured at 600, 532, and 450 nm. The concentration of MDA was calculated according to Heath and Packer (1968).

Electrolyte leakage (EL) was measured with an electrical conductivity meter (FE38, Mettler Toledo, Switzerland) according to Lutts et al. (1996). Briefly, rice samples were washed with deionized water three times and cut into 2-cm pieces. Then, the samples were placed in tubes containing 10 ml water and incubated on a shaker at 30°C for 6 h. After incubation, electrical conductivity was measured as EC1. The tubes were further incubated in boiling water for 30 min, and the electrical conductivity was measured as EC2. EL was calculated as EC1/EC2.

### Antioxidant Enzymes Activities

Fresh root and leaf samples were homogenized with 3 ml sodium phosphate buffer (50 mM, pH = 7.8) containing 1 mM ethyl diamine tetraacetic acid and 2% (*w*/*v*) polyvinyl pyrrolidone. The raw extract was centrifuged at 4°C for 20 min at 10,000 rpm, and the supernatant was used for the measurements of soluble protein concentration and antioxidant enzyme activity. Superoxide dismutase (SOD) activity was determined following the protocol of Giannopolitis and Ries (1977), and 1 U was defined as 50% inhibition of nitro blue tetrazolium reduction. Ascorbate peroxidase (APX) activity was determined using the procedure described by Nakano and Asada (1981). Catalase (CAT) activity was measured according to Cakmak and Marschner (1992). Polyphenoloxidase (POD) activity assay was conducted with the method described by Liang et al. (2003).

### Tissue Sap Osmotic Potential and Osmolytes Concentrations

Fresh samples were placed into 0.5-ml tubes and frozen in liquid nitrogen for 30 min. Then, the tubes were drilled at the bottom and put into 1.5-ml tubes without lid until plant samples thawed. Tissue sap was collected through centrifugation at 3,000 rpm for 10 min, and 10 μl tissue sap was used to measure osmotic potential with a dew point osmolarity meter.

The concentrations of osmolytes including proline, soluble sugar, and soluble protein were analyzed. Soluble protein was measured spectrophotometrically at 595 nm using the method described by Bradford (1976) with Coomassie brilliant blue G-250 and calculated with a standard curve of bovine serum albumin. Proline concentration was assayed according to Bates et al. (1973). Fresh samples were homogenized with 3% (*w/v*) aqueous sulfosalicylic acid and incubated in boiling water for 10 min. Then, 1 ml extract was mixed with 1 ml glacial acetic acid and 1 ml ninhydrin solution. The mixture was incubated at 100°C for 1 h and cooled to room temperature; then, 5 ml toluene was added and mixed thoroughly. The absorbance of the upper phase was measured at 520 nm, and proline concentration was calculated with a standard curve. Soluble sugar was extracted from the powder of dry sample three times with 3 ml 80% (*v/v*) ethanol at 80°C, and the supernatant was collected after centrifugation at 10,000 rpm for 10 min each time. The extracts were mixed, and the total volume was adjusted to 10 ml. Soluble sugar concentration was measured with the anthrone-sulfuric acid method and calculated with a sucrose standard curve.

### Aquaporin Gene Expression

For aquaporin gene expression analysis, rice seedlings were pretreated with or without 1.5 mM silicic acid for 3 days before NaCl or PEG was added. After 6 or 24 h of treatment, rice root samples were harvested and saved in liquid nitrogen. Total RNA was extracted using a plant RNA extraction kit (MiniBEST, Takara, Japan) and treated with DNase I in the kit to remove genomic DNA. The isolated RNA was then converted to complementary DNA (cDNA) using a prime script RT reagent kit (Takara, Japan). The expression of aquaporin genes (including *OsPIP1;1*, *OsPIP1;2*, *OsPIP2;1*, *OsPIP2;2*, *OsPIP2;4*, and *OsPIP2;6*) was determined by real-time quantitative PCR using TB Green premix Ex Taq (Takara, Japan) on a Lightcycler 480II system (Roche Diagnostics, Switzerland) following manufacturer’s instructions. *OsActin1* was used as the internal reference. The sequences of primers used in this study are listed in Supplementary Table S1.

### Statistical Analysis

All the data in this research were calculated with Excel (Microsoft, United States) and subjected to two-way analysis of variance (two-way ANOVA). Significant difference was determined with *P* < 0.05 [least significant difference (LSD)]. Plant growth data (a composite of DW, *Pn*, *Tr*, chlorophyll, MDA, EL, and RWC for the shoot, and a composite of DW, root/shoot ratio, total root length, total root surface area, root number, MDA, EL, and RWC for the root) were converted into a dissimilarity matrix using the Euclidean distances without standardization. For spatial ordinations, principal coordinate analysis (PCoA) was performed using the cmdscale function in “vegan” package of R (version 3.5.0) (R Core Team, 2014; Oksanen et al., 2019).

## Results

### Effects of NaCl and Iso-Osmotic PEG on Plant Growth

In rice shoot, NaCl and PEG decreased DW by 20.12 and 15.02%, respectively. The MDA concentration and EL of rice shoot were higher under NaCl treatment than under PEG treatment. However, there was no significant difference between NaCl- and PEG-induced decrement of *Pn*, *Tr*, chlorophyll concentration, and RWC in rice shoot (Table 1). In rice root, NaCl and PEG decreased DW by 43.12 and 22.36%, respectively. In addition, NaCl treatment caused more severe total length and surface area decline, MDA accumulation, and EL increment in rice root than those under PEG treatment (*P* < 0.05). However, there was no significant difference between root water status decline caused by NaCl or PEG (Table 2). Together, NaCl caused more severe growth limitation and damages in rice than PEG.

**TABLE 1:** Shoot dry weight (DW), photosynthetic rate (*Pn*), transpiration rate (*Tr*), chlorophyll concentration, shoot malondialdehyde (MDA) concentration, shoot electrolyte leakage (EL), and shoot relative water content (RWC) of rice under different treatments. Alleviation effects of Si on these parameters in rice under NaCl or PEG treatments.

**Parameter**	**Control**	** + Si**	**NaCl**	**NaCl + Si**	**PEG**	**PEG + Si**	**Alleviation effect of Si**	
							**NaCl**	**PEG**
Shoot DW (mg)	380 ± 15a	383 ± 22a	303 ± 13b	370 ± 17a	323 ± 6b	365 ± 11a	+17.6%	+11.2%
*Pn* (μmol CO_2_ m^2^ s^–1^)	11.5 ± 0.8a	10.9 ± 0.3a	5.93 ± 0.60c	8.05 ± 0.66b	6.27 ± 0.53c	7.90 ± 0.33b	+18.4%	+14.1%
*Tr* (mmol H_2_O m^–2^ h^–1^)	2.50 ± 0.26a	2.39 ± 0.24a	1.11 ± 0.19c	1.69 ± 0.14b	1.23 ± 0.15c	1.61 ± 0.17b	+23.2%	+15.2%
Chlorophyll (mg g^–1^ FW)	3.75 ± 0.13ab	3.92 ± 0.14a	3.03 ± 0.08c	3.50 ± 0.13b	3.23 ± 0.07c	3.81 ± 0.10a	+12.5%	+15.5%
MDA (nmol g^–1^ FW)	8.99 ± 0.78c	6.28 ± 0.32c	22.5 ± 2.9a	11.0 ± 1.9c	16.0 ± 2.0b	9.84 ± 1.45c	−127%	−68.7%
EL (%)	16.3 ± 1.8c	16.6 ± 3.0c	54.8 ± 3.5a	22.5 ± 1.4b	28.2 ± 3.2b	17.6 ± 1.5c	−198%	−64.8%
RWC (%)	82.9 ± 1.5a	82.7 ± 1.2a	75.2 ± 1.5c	78.5 ± 1.4b	76.9 ± 1.2c	80.7 ± 0.8ab	+3.22%	+3.88%

*Rice seedlings were grown hydroponically with control, 100 mM NaCl (NaCl), and 18% (*w*/*v*) PEG-6000 (PEG) in the absence or presence (+ Si) of 1.5 mM Si for 5 days. Shoot growth parameters are shown as mean values ± SE of three independent biological replicates, and different letters indicate significant differences (*P* < 0.05). The alleviation effects of Si were calculated using mean values.*

**TABLE 2:** Root dry weight (DW), root/shoot ratio, total root length, root surface area, root number, root malondialdehyde (MDA) concentration, root electrolyte leakage (EL), and root relative water content (RWC) of rice under different treatments. Alleviation effects of Si on these parameters in rice under NaCl or PEG treatments.

**Parameter**	**Control**	** + Si**	**NaCl**	**NaCl + Si**	**PEG**	**PEG + Si**	**Alleviation effect of Si**	
							**NaCl**	**PEG**
Root DW (mg)	81.1 ± 2.6a	83.4 ± 4.1a	46.1 ± 3.6d	67.4 ± 4.0bc	63.0 ± 3.0c	74.7 ± 3.4ab	+26.2%	+14.5%
Root/shoot ratio (%)	21.4 ± 1.1a	21.8 ± 1.2a	15.2 ± 0.5c	18.3 ± 2.0b	19.5 ± 0.7ab	20.5 ± 2.0*a*b	+14.4%	+4.39%
Total length (cm)	401 ± 31ab	447 ± 57a	196 ± 7d	312 ± 33c	279 ± 38c	380 ± 18b	+31.3%	+25.2%
Surface area (cm^2^)	29.3 ± 2.8a	32.0 ± 3.7a	10.4 ± 1.2d	16.1 ± 0.4c	17.1 ± 1.6c	23.3 ± 2.1b	+19.5%	+21.2%
Root number	333 ± 17ab	407 ± 36a	176 ± 20d	267 ± 39b	243 ± 16c	326 ± 18b	+27.4%	+25.0%
MDA (nmol g^–1^ FW)	7.59 ± 0.30c	5.66 ± 0.37c	16.9 ± 1.9a	6.68 ± 1.29c	11.4 ± 0.9b	6.24 ± 0.68c	−160%	−67.9%
EL (%)	41.4 ± 3.1c	40.2 ± 1.3c	67.4 ± 3.8a	57.3 ± 3.1b	57.1 ± 4.4b	41.6 ± 1.2c	−24.3%	−37.7%
RWC (%)	82.8 ± 1.3a	81.7 ± 0.9a	76.0 ± 0.8b	81.7 ± 2.6a	77.8 ± 1.2b	83.5 ± 0.9a	+5.64%	+6.81%

*Rice seedlings were grown hydroponically with control, 100 mM NaCl (NaCl), and 18% (*w*/*v*) PEG-6000 (PEG) in the absence or presence (+ Si) of 1.5 mM Si for 5 days. Root growth parameters are shown as mean values ± SE of three independent biological replicates, and different letters indicate significant differences (*P* < 0.05). The alleviation effects of Si were calculated using mean values.*

### Effects of Si on Plant Growth Under NaCl and Iso-Osmotic PEG

Under control condition, Si addition had no obvious effect on rice seedling growth (Tables 1, 2). However, in rice shoot, Si addition alleviated the NaCl-induced decline of DW, *Pn*, *Tr*, and chlorophyll concentration (Table 1). Silicon also promoted root growth under salt stress with higher root DW and better root morphological traits (Table 2). In addition, exogenous Si significantly improved RWC and decreased MDA concentration and EL in both the shoot and root (*P* < 0.05, Tables 1, 2). The growth promotion effect of Si was also observed under PEG treatment but differed significantly from that under NaCl. Si improved shoot DW by 17.58 and 11.24% and improved root DW by 26.23 and 14.50% under NaCl and PEG, respectively. In rice shoot, *Pn*, *Tr*, and chlorophyll concentration were improved by Si addition by 18.39, 23.20, and 12.53% under NaCl, and by 14.14, 15.20, and 15.47% under PEG, respectively (Table 1). In rice root, Si addition promoted total root length, root surface area, and root number under NaCl and PEG at similar levels (Table 2). Under both NaCl and PEG treatments, Si addition decreased MDA concentration and EL significantly (*P* < 0.05) in rice shoot and root, while the alleviation effects on oxidative damages were more significant under NaCl than those under PEG. Moreover, the promotion effect of Si on RWC under NaCl was close to that under PEG in both the shoot and root (Tables 1, 2).

**FIGURE 1:**
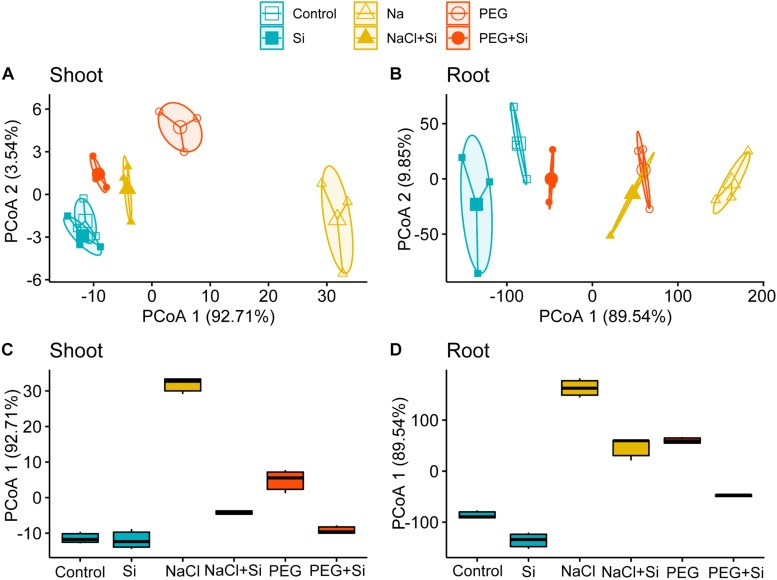
Principal coordinate analysis (PCoA) of **(A)** shoot and **(B)** root growth parameters. Boxplots for visualizing the Euclidean distance of the first axis of PCoA of **(C)** shoot and **(D)** root growth parameters. Shoot and root growth parameters were measured after a 5-day treatment of control, 100 mM NaCl (NaCl), and 18% (*w/v*) PEG-6000 (PEG) in the absence (–Si) or presence (+ Si) of 1.5 mM Si (shoot and root growth parameters are shown in Tables 1, 2, respectively).

To parse the growth promotion effects of Si in response to ionic and osmotic components of salt stress comprehensibly, PCoA was conducted to compare the growth parameters of rice root or shoot under different treatments in Tables 1, 2. The results showed that the first principal axis accounts for 92.71 and 89.54% variation of shoot and root growth parameters, respectively (Figures 1A,B). Box plots were further plotted based on the data mapped on the first axis of PCoA to visualize the effects of stress conditions and Si addition (Figures 1C,D). The distance between NaCl and control was larger than that between PEG and control in both the shoot and root, and the corresponding distance became smaller when Si was added (Figures 1C,D). However, the Si-induced distance change under NaCl treatment was larger than that under PEG treatment in rice shoot, while the Si-induced distance changes under NaCl and PEG treatments were at an equal level in root (Figures 1C,D).

### Effects of Si on Na and K Concentrations

**FIGURE 2:**
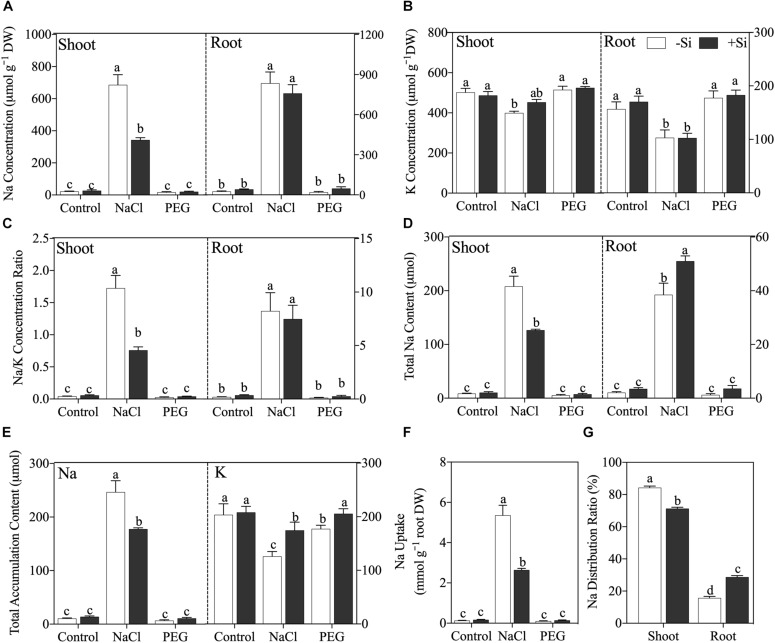
**(A)** Na concentration, **(B)** K concentration, **(C)** Na/K ratio, **(D)** Na accumulation content in shoot and root, **(E)** plant total Na and K accumulation content, **(F)** Na uptake, and **(G)** Na distribution ratio. Rice were grown hydroponically with control, 100 mM NaCl (NaCl), and 18% (*w/v*) PEG-6000 (PEG) in the absence (−Si, white column) or presence (+ Si, black column) of 1.5 mM Si for 5 days. The data are the mean values ± SE of three independent biological replicates, and different letters show significant differences (*P* < 0.05).

After a 5-day treatment, NaCl caused significant Na accumulation and disrupted Na/K balance in both the shoot and root, while non-ionic osmotic stress (PEG) induced no obvious effect on Na and K in rice seedling except a decline of plant total K accumulation content (Figure 2). Under NaCl treatment, the addition of Si significantly decreased shoot Na concentration (*P* < 0.05) but not in rice root (Figure 2A). NaCl treatment also decreased K concentration in both the shoot and root, but Si addition had no obvious effect on K concentration in either the shoot or root (Figure 2B). NaCl treatment raised Na/K ratio in both the shoot and root, while Si addition alleviated the increment of Na/K ratio in the shoot but not in the root (Figure 2C). In addition, exogenous Si increased Na accumulation in the root, while it decreased that in the shoot (Figure 2D). As for total Na and K accumulation content in rice seedling, NaCl treatment increased total Na content and decreased total K content, which was alleviated by Si addition (Figures 2E,F). Si also ameliorated total K content decline, which was induced by PEG treatment (Figure 2E). Moreover, Si decreased Na uptake rate (Figure 2F) and altered Na distribution between the shoot and root with more Na sequestered in the root under NaCl treatment (Figure 2G).

### Effects of Si on Tissue Osmotic Potential and Osmolyte Concentrations

**FIGURE 3:**
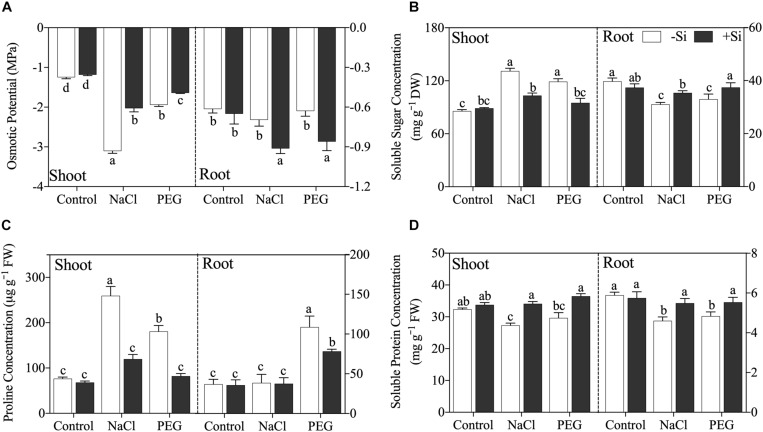
**(A)** Tissue sap osmotical potential, **(B)** soluble sugar concentration, **(C)** proline concentration, and **(D)** soluble protein concentration in shoot and root. Rice were grown hydroponically with control, 100 mM NaCl (NaCl), and 18% (*w/v*) PEG-6000 (PEG) in the absence (−Si, white column) or presence (+ Si, black column) of 1.5 mM Si for 5 days. The data are the mean values ± SE of three independent biological replicates, and different letters show significant differences (*P* < 0.05).

Exogenous Si significantly increased shoot sap osmotic potential but decreased root sap osmotic potential under both NaCl and PEG treatments (Figure 3A). As for compatible osmolytes, both NaCl and PEG treatments increased the concentrations of soluble sugar and proline and decreased soluble protein concentration in rice shoot, and decreased soluble sugar and soluble protein in rice root (Figures 3B–D). However, proline concentration was not affected by NaCl but increased significantly by PEG in rice root (Figure 3C). Si addition decreased the concentration of soluble sugar in the shoot but increased that in the root (Figure 3B). In addition, proline concentration was decreased by Si addition in the shoot of rice under both NaCl and PEG treatments. However, Si addition only decreased the concentration of proline in the root under PEG treatment but not in that under NaCl treatment (Figure 3C). Moreover, soluble protein was improved significantly by Si in both the shoot and root of rice under NaCl and PEG treatments (Figure 3D).

### Effects of Si on Antioxidant Enzymes Activities

**FIGURE 4:**
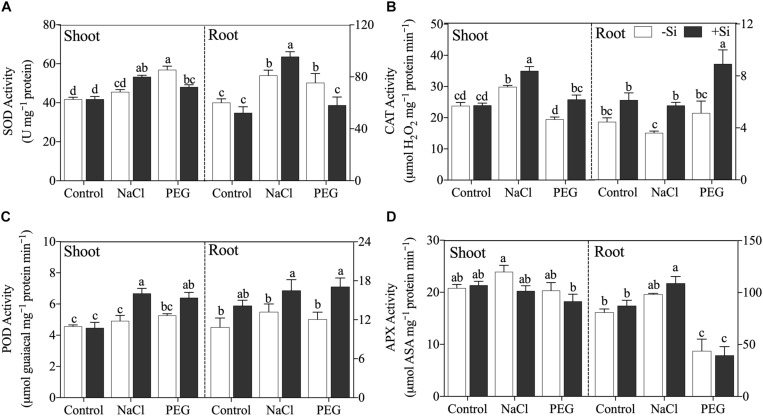
**(A)** Superoxide dismutase (SOD) activity, **(B)** catalase (CAT) activity, **(C)** polyphenoloxidase (POD) activity, and **(D)** ascorbate peroxidase (APX) activity in shoot and root. Rice were grown hydroponically with control, 100 mM NaCl (NaCl), and 18% (*w/v*) PEG-6000 (PEG) in the absence (−Si, white column) or presence (+ Si, black column) of 1.5 mM Si for 5 days. The data are the mean values ± SE of three independent biological replicates, and different letters show significant differences (*P* < 0.05).

Generally, Si addition changed the activity of antioxidant enzyme and the regulation effects of Si differed with stress conditions (Figure 4). Si addition increased SOD activity under NaCl treatment but decreased SOD activity under PEG treatment in both the shoot and root (Figure 4A). Si application enhanced CAT and POD activities in the shoot and root under both NaCl and PEG treatments significantly (Figures 4B,C), while APX activity was not altered by the addition of Si under both stress conditions (Figure 4D).

### Effects of Si on the Expression of Aquaporin Genes

**FIGURE 5:**
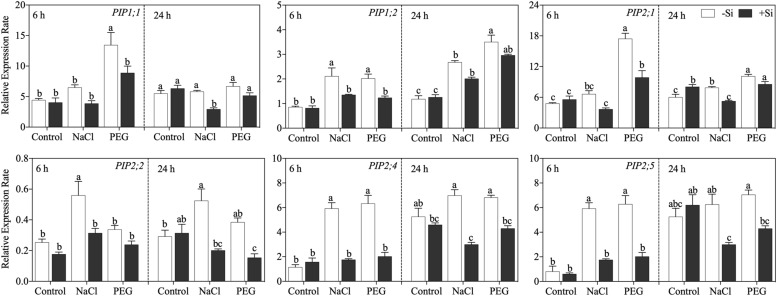
Expression of *OsPIP1;1*, *OsPIP1;2*, *OsPIP2;1*, *OsPIP2;2*, *OsPIP2;4*, and *OsPIP2;6* in root. Rice seedlings were pretreated with (+ Si, black column) or without (−Si, white column) 1.5 mM Si for 3 days and then grown under control, 100 mM NaCl (NaCl), and 18% (*w/v*) PEG-6000 (PEG) for 6 or 24 h. The data are the mean values ± SE of three independent biological replicates, and different letters show significant differences (*P* < 0.05).

The expressions of six known members of the *PIP* gene family at 6 and 24 h after treatment were analyzed. The effects of Si on aquaporin gene expression varied among genes and stress conditions (Figure 5). In detail, after 6 h of treatment, NaCl treatment upregulated the expression of *OsPIP1;2*, *OsPIP2;2*, and *OsPIP2;4*. PEG treatment upregulated the expression of *OsPIP1;1*, *OsPIP1;2*, *OsPIP2;1*, *OsPIP2;4*, and *OsPIP2;6* with the exception of*OsPIP2;2*. Exogenous Si addition markedly downregulated the expression of these *PIP* genes under NaCl and PEG treatments. After 24 h of treatment, the expressions of *OsPIP1;2*, *OsPIP2;1*, and *OsPIP2;2* were still increased by both NaCl and PEG treatments but were decreased by Si application. Moreover, the inhibition effect of Si also occurred at 24 h for *OsPIP2;4*. NaCl treatment reduced the expression of *OsPIP2;6*, while no significant effect of Si addition was observed.

## Discussion

Salt stress is one of the most common environmental problems. It has been estimated that over 20% arable land is subject to salinity stress (Munns and Tester, 2008; Munns and Gilliham, 2015). Rice is an important staple food crop that is sensitive to salt stress (Gong et al., 2006; Shi et al., 2013). Salt stress can cause rice growth limitation and yield decline, and severe salt stress would even result in plant death, which would threaten stable food supply globally (Zhu and Gong, 2014; Munns and Gilliham, 2015). Si is effective in improving the growth of plant under salinity, which imposes ionic toxicity and osmotic constraint on plant (Liang et al., 2007; Zhu and Gong, 2014; Coskun et al., 2016). In this study, we used iso-osmotic growth substances developed from NaCl and PEG to distinguish the effects of Si in response to osmotic and ionic components of salinity stress. Interestingly, the comparison between the growth promotion effects of Si under NaCl and PEG shows Si alleviated ionic and osmotic components of salinity stress in rice differently between shoot and root. We further evaluated the role of Si in ionic homeostasis and water status maintenance in rice under salinity and found that Si can alleviate ionic toxicity and water constraint in rice in an organ-specific pattern and promote rice salinity tolerance at the whole plant level.

### Si Alleviated Salinity Stress in Rice in an Organ-Specific Pattern

It is well-documented that both ionic and osmotic components can severely limit the growth of plant under salinity (Munns, 2002; Munns and Tester, 2008). Because of the additional ionic component, we observed more severe growth limitation and stress damages in rice under salinity than under iso-osmotic PEG-derived osmotic stress in this study (Tables 1, 2). In line with previous researches concerning the effects of Si under salinity or osmotic stresses in rice (Ming et al., 2012; Shi et al., 2013), exogenous Si promoted the growth of rice seedlings under both NaCl and iso-osmotic PEG conditions (Tables 1, 2). However, the growth promotion effects of Si in rice were more significant under NaCl treatment than under PEG treatments, indicating that Si can alleviate both ionic and osmotic components of salinity stress. Therefore, it is feasible to investigate the role of Si in alleviating ionic toxicity and osmotic constraint by comparing the effects of Si under NaCl and PEG treatments.

The strategy used in previous researches to distinguish the effects of ionic and osmotic components of salinity on plant metabolism (e.g., K homeostasis, polyamine metabolism, and transcription) is to subtract osmotic effect from total effects of salinity (Shabala, 2000; Lefevre et al., 2001; Umezawa et al., 2002). However, when the effect of Si on plant growth in response to ionic and components of salinity is assessed, it is unreliable to evaluate plant growth status using single growth parameter (e.g., DW). Therefore, we conducted PCoA to compare the effects of Si in rice shoot and root under NaCl and iso-osmotic PEG with different kinds of growth parameters involved (Tables 1, 2). Consistent with the alleviation effects of Si in shoot and root under salt stress, the distance between control and NaCl treatment in PCoA was decreased by the addition of Si (Figures 1C,D). In addition, it is clearly shown in Figure 1 that Si could promote rice growth under salt stress through alleviating both ionic and osmotic components of salinity stress in the shoot but mainly osmotic constraint in the root. Based on the differences in growth promotion effects of Si on ionic and osmotic components between the shoot and root, it can be concluded that Si alleviates salt stress in an organ-specific pattern in rice.

### Si Alleviated Ionic Toxicity by Regulating Na Distribution

Ionic homeostasis is essential for the plant exposed to excess Na (Munns, 2002; Munns and Tester, 2008). Under salinity, Na is inevitably absorbed following transpiration flow and accumulates in plant. Given the fact that K is closely coupled to enzyme activation and protein synthesis in plant (Wang and Wu, 2013), the competition between Na and K for binding sites of transport proteins and enzymes can lead to K concentration decline and successive metabolism disruption in plant (Kronzucker and Britto, 2011). In this study, Si addition decreased Na concentration remarkably and increased K concentration (although not significantly) in rice shoot, which would protect rice from Na toxicity. However, no obvious effect of Si on Na and K concentrations was observed in rice root (Figure 2); this is in accordance with the result of PCoA that Si cannot alleviate ionic toxicity in rice root.

When subject to salinity stress, the shoot is more sensitive to Na than the root (Munns, 2002); therefore, it is of pivotal importance to lower shoot Na accumulation with respect to plant salt tolerance (Munns and Tester, 2008). Although the concentration of Na in rice root was not changed by Si addition, total Na accumulation content in the root was enhanced by Si (Figure 2) because of the Si-induced root biomass increment (Table 2). Therefore, Si addition induced more Na compartmented in rice root, which can protect the shoot from Na toxicity. However, Bosnic et al. (2018) found that Si addition increased Na root-to-shoot translocation by regulating the expression of genes related to Na xylem loading and unloading in maize. The different effects of Si on Na translocation between rice and maize indicate the mechanism of Si-induced ionic detoxification differs with plant species. Moreover, Si decreased plant total Na accumulation content by decreasing net Na uptake rate (Figure 2), which could be due to the apoplastic blockage effect of Si on Na transport (Gong et al., 2006; Flam-Shepherd et al., 2018). In summary, our results imply that Si can regulate Na distribution between the shoot and root, thereby alleviating Na toxicity under salinity at the whole plant level.

### Si Alleviated Osmotic Constraint by Improving Water Uptake and Transport

Maintaining optimal water status by improving root water uptake and decreasing water loss is a major strategy for plant in the adaptation to salinity stress (Steudle, 2000a, b; Aroca et al., 2012). Previous research in maize showed that Si alleviated water deficiency by reducing transpiration rate and water loss (Gao et al., 2006). By contrast, in this study, rice transpiration rate was decreased by salinity, while Si addition increased transpiration rate (Table 1), which would lead to more water loss. This result implies that water uptake promotion but not water loss decline could play a dominant role in Si-induced water status promotion in rice under salinity in accordance with the suggestion by Chen et al. (2018).

In plant root, water uptake capacity mainly depends on root–water contact area, water uptake driving force, and root water permeability (Steudle, 2000a; Vandeleur et al., 2009; Sutka et al., 2011). Under water-deficit conditions (e.g., salinity stress and drought stress), root growth is significantly limited (Javot and Maurel, 2002; Farooq et al., 2009). In this study, Si increased total root length and total root surface area (Table 2), thereby improving root–water contact area. Besides, Si addition also decreased root sap osmotic potential (Figure 3A) through regulating the concentrations of soluble sugar and soluble protein in rice root (Figures 3B,D), thus leading to more water uptake via enhanced water uptake driving force. Moreover, it has been reported that Si can increase root water permeability through upregulating aquaporin gene expression in sorghum under water-deficit conditions (Liu et al., 2014, 2015). However, in this study, NaCl and PEG treatments upregulated *OsPIP*s expression, while Si decreased the expression of aquaporin genes under both NaCl and PEG treatments (Figure 5). Similar effects of stress condition and Si addition on the expression of *PIP*s have been reported in salt-stressed cucumber (Zhu et al., 2015) and drought-stressed tomato (Shi et al., 2016). These results indicate that the effects of Si on the expression of aquaporin genes differ with plant species. In rice, the upregulated expression of *OsPIP*s could be an adaptation strategy to disrupted water status. In this study, Si addition promoted rice water status under NaCl and PEG treatments by increasing root–water contact area and osmotic force; consequently, the expression of *OsPIP*s remained unchanged relative to control treatment.

After uptake by the root, water is transported to the shoot driven by a combination of shoot osmotic force and transpiration force. Although the Si-improved transpiration rate may lead to more water loss, it can also drive more water transport from the root to the shoot. It is worth noting that the regulation effect of Si on osmotic potential and osmolytes in rice shoot was different from that in rice root. Si increased osmotic potential and decreased the concentrations of soluble sugar and proline in rice shoot (Figure 3). Given the high energy cost in osmolytes synthesis (e.g., soluble sugar and proline), the different Si-induced osmotic regulation strategies between the shoot and root imply that Si can regulate root water uptake and successively protect the shoot from excess energy cost in osmolytes metabolism (Raven, 1985). Considering the converse trends of compatible solute concentration between the shoot and the root in this study (Figure 3), we speculate that the Si-regulated osmolytes transport from the shoot to the root could be the underlying foundation of the regulation effect of Si on osmolytes concentrations, which is consistent with Zhu et al. (2016) who has reported that Si can increase shoot-to-root soluble sugar transport in salt-stressed cucumber. The transport of osmolytes could successively provide energy for root growth and contribute to root osmotic potential adjustment. Taken together, Si could promote root-to-shoot water transport via increasing transpiration force and root water supply.

In addition, both ionic and osmotic stresses can cause membrane oxidative damage in plant (Zhu, 2001; Munns, 2002; Tables 1, 2). Oxidative damages would disrupt the functions of plasma membrane and further influence ion balance and water uptake in plant under salinity (Zhu and Gong, 2014; Coskun et al., 2016). In the present study, Si was effective in alleviating oxidative damages through regulating antioxidant enzymes such as SOD, CAT, POD, and APX under salinity. This is consistent with previous studies of oxidative damage in plant (Liang et al., 2003; Zhu et al., 2004). In addition, our results showed that exogenous Si could modulate these antioxidant enzymes distinctly in response to ionic and osmotic components (Figure 4). The beneficial effect of Si on oxidative damages contributes to ionic detoxification and water status maintenance in both the shoot and root. In the root, the Si-induced membrane stabilization can promote water uptake and regulate Na transport. However, in rice shoot, Si can maintain photosynthesis and metabolism to improve plant growth by regulating antioxidant enzymes and alleviating oxidative damages.

## Conclusion

**FIGURE 6:**
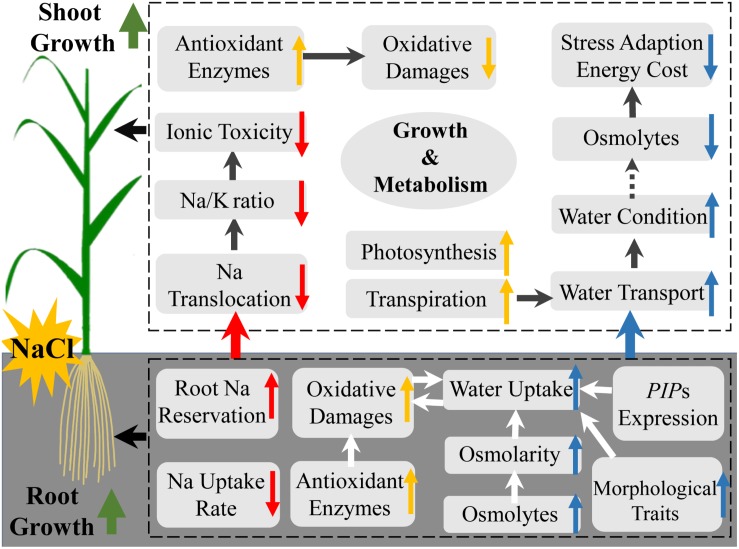
A schematic model of the mechanisms of silicon-induced salt stress resistance in rice in relation to ionic detoxification (red arrow), water status maintenance (blue arrow), oxidative damage alleviation, and other growth regulation (yellow arrow).

Our results indicate that Si improves salt stress tolerance in rice by alleviating ionic toxicity and osmotic constraint in an organ-specific pattern (Figure 6). Si ameliorates ionic toxicity by decreasing Na uptake and root-to-shoot translocation. Si alleviates osmotic constraint by regulating root morphological traits and osmotic potential but not aquaporin-related gene expression for water uptake, and promoting transpiration force but not osmotic force in the shoot for root-to-shoot water transport (Figure 6).

## Data Availability Statement

All datasets generated for this study are included in the article/Supplementary Material.

## Author Contributions

GY and YL designed the study and wrote the manuscript. GY, XF, MP, CY, and ZX conducted the experiment and analyzed the data.

## Conflict of Interest

The authors declare that the research was conducted in the absence of any commercial or financial relationships that could be construed as a potential conflict of interest.
