# Supplementary material for: Silicon Improves Rice Salinity Resistance by Alleviating Ionic Toxicity and Osmotic Constraint in an Organ-Specific Pattern
Source: Front Plant Sci. 2020 Mar 12;11:260. doi: 10.3389/fpls.2020.00260 (PMC7081754; doi:10.3389/fpls.2020.00260)
Supplement: Supplementary file 1 [file Table_1.DOCX]

**Table S1** Primers used in this study and related references

| Gene | Accession number | Primer sequence (5’-3’) | Product length | Reference |
| --- | --- | --- | --- | --- |
| *OsPIP1;1* | AK061769 | F: ATCTTCTGGGTTGGTCCCTTCGTT | 141 bp | (Meng et al., 2016) |
|  |  | R: ATCACGATTGCGTTGCATGTCGTC |  |  |
| *OsPIP1;2* | AK098849 | F: ATGCCTGGGATGACCATTGGATCT | 120 bp | (Meng et al., 2016) |
|  |  | R: ATGCAGGTTACGACCTGCTCTTGA |  |  |
| *OsPIP2;1* | AK072519 | F: GCTGGAAGGCGTTGATGAAGCAAT | 143 bp | (Meng et al., 2016) |
|  |  | R: ACTTCACACACACGACAAGCAGGA |  |  |
| *OsPIP2;2* | AK061782 | F: CCCAATTGGATTCGCGGTGTTCAT | 121 bp | (Meng et al., 2016) |
|  |  | R: ATCCCAGGCCTTGTCCTTGTTGTA |  |  |
| *OsPIP2;4* | AK072632 | F: CAACAACAACAAGGCCTGGAGTGA | 192 bp | (Sakurai et al., 2005) |
|  |  | R: GAAAGAGCCCAAACAATGCCGACT |  |  |
| *OsPIP2;6* | AK061312 | F: CCTGGTTGGACTTGGTCATATCGT | 89 bp | (Meng et al., 2016) |
|  |  | R: ACAAATCATGCACCTGGCTGACTG |  |  |
| *OsActin* | AB047313 | F: TTATGGTTGGGATGGGACA | 197 bp | (Bañuelos et al., 2002) |
|  |  | R: AGCACGGCTTGAATAGCG |  |  |

Bañuelos, M.A., Garciadeblas, B., Cubero, B., and Rodríguez-Navarro, A. (2002). Inventory and functional characterization of the HAK potassium transporters of rice. *Plant Physiol.* 130(2)**,** 784–795. doi: 10.1104/pp.007781.

Meng, D.L., Walsh, M., and Fricke, W. (2016). Rapid changes in root hydraulic conductivity and aquaporin expression in rice (*Oryza sativa* L.) in response to shoot removal - xylem tension as a possible signal. *Ann. Bot.* 118(4)**,** 809–819. doi: 10.1093/aob/mcw150.

Sakurai, J., Ishikawa, F., Yamaguchi, T., Uemura, M., and Maeshima, M. (2005). Identification of 33 rice aquaporin genes and analysis of their expression and function. *Plant Cell Physiol.* 46(9)**,** 1568–1577. doi: 10.1093/pcp/pci172.
